# Latin America and Caribbean's path to improve hypertension control: time for bolder, tougher actions

**DOI:** 10.1016/j.lana.2022.100278

**Published:** 2022-05-14

**Authors:** The Lancet Regional Health– Americas

High blood pressure (BP) is the most important reversible risk factor for cardiovascular diseases (CVD), the leading cause of death and disability in the Americas. Screening for high BP takes less than 15 min and requires very little: a chair, a validated BP measurement device, and one trained health-care professional. If identified, high BP can be controlled with effective and relatively cheap medicines. Hypertension is defined as BP ≥140/90 mmHg (or receiving antihypertensive drug treatment) and improving hypertension control, including among those at high risk (<130 mmHg systolic BP), is paramount to reduce deaths and prevent CVD-related events. Yet, in Latin America and the Caribbean (LAC) region, rates of hypertension diagnosis are still suboptimal, and only 35% of women and 23% of men with diagnosed hypertension have their BP under control.

The lack of awareness about hypertension and its consequences, however, is not restricted to the Americas. Worldwide, the number of people aged 30–79 years with hypertension doubled from 1990 to 2019, despite our increasing knowledge of the topic. The issue has received a fair amount of attention over the past decades—at least from researchers, medical associations, and non-governmental agencies. In 2016, the *Lancet* Commission on Hypertension published a call-for-action statement with ten essential and achievable goals and ten key actions to improve hypertension detection, treatment, and control globally. In the same year, WHO created *The Global HEARTS Initiative*, to support countries to implement actions to prevent CVD. Likewise, the Pan American Health Organization, together with partner organisations and ministries of health, created the *HEARTS in the Americas Initiative*, a regional adaptation of the WHO HEARTS Initiative, with the focus on enhancing hypertension management and CVD secondary prevention—the key issue in the Americas region. Indeed, a recent time-trend analysis by the NCD Risk Factor Collaboration highlighted a modest improvement in hypertension care in many middle-income countries, including some in Latin America. Costa Rica, for example, now outperforms most high-income nations for high BP awareness (70–80%), treatment (60–76%), and control (45–55%).

Despite the many advances observed, the LAC region still holds unacceptably high rates of undiagnosed hypertension, especially among younger people, surpassing 50% for men aged 30–44 years. These underscore the need to implement and expand primary care programmes to increase awareness and screening for high BP. Two important limitations that require careful consideration include the lack of access to primary care (including medicines) and the indiscriminate use of unvalidated BP measurement devices. Although relatively straightforward, accurate BP measurement requires access to validated devices, professional training, and attention to details—such as use of the correct cuff size, placement of the cuff in bare arms, arm support at heart level, and so on). Securing access to scientifically validated, low-cost automated devices, ideally adapted for use in low-resource communities (eg, solar-powered devices to attend powerless areas and isolated communities), should be a priority in places with low rates of diagnosis such as Peru, where only 35% of men with hypertension have a reported diagnosis. In response to the unmet need to improve hypertension care in the LAC region, the *HEARTS in the Americas initiative* published a new recommendation in this issue of the *Lancet Regional Health – Americas*. The multidisciplinary study group identified eight evidence-based “drivers” of better hypertension control (ie, interventions) and “scorecards” to help improve hypertension control in primary care practice. In 2021, WHO released an updated guideline for the pharmacological treatment of hypertension in adults. Campbell and colleagues discussed how the new guideline can be integrated with other global and regional technical documents in the LAC region while also calling health advocates and policy makers to “*prioritize the prevention and control of hypertension to improve the health and wellbeing of their populations and reduce CVD health disparities within and between populations of the Americas*”.

Noticeably, the one thing all those statements, call-for-actions, and initiatives have in common is that improvement of hypertension care will require increased health-care use and expanded primary care access, with priority for team-based care. Strengthening CVD prevention and hypertension care provides an opportunity to reinforce the importance of advocating for accessible universal health-care coverage.

Unlike some emerging diseases and chronic conditions with no preventive or therapeutic means, we already have the knowledge and the treatments required to control hypertension and prevent CVD events and deaths. The challenge, in this case, is to implement effective and sustained primary-care approaches that include BP control as a priority and secure long-lasting access to care for everyone. We already have the cards, now we must use them.

